# Reduced Antibody Acquisition with Increasing Age following Vaccination with BNT162b2: Results from Two Longitudinal Cohort Studies in The Netherlands

**DOI:** 10.3390/vaccines10091480

**Published:** 2022-09-06

**Authors:** Lotus Leonie van den Hoogen, Mardi Boer, Abigail Postema, Lia de Rond, Mary-lène de Zeeuw-Brouwer, Inge Pronk, Alienke Jentien Wijmenga-Monsuur, Elske Bijvank, Caitlyn Kruiper, Lisa Beckers, Marjan Bogaard-van Maurik, Ilse Zutt, Jeffrey van Vliet, Rianne van Bergen, Marjan Kuijer, Gaby Smits, W. M. Monique Verschuren, H. Susan J. Picavet, Fiona Regina Maria van der Klis, Gerco den Hartog, Robert Samuel van Binnendijk, Anne-Marie Buisman

**Affiliations:** 1 Centre for Immunology of Infectious Diseases and Vaccines, National Institute for Public Health and the Environment (RIVM), 3721 MA Bilthoven, The Netherlands; 2 Centre for Prevention and Health Services Research, National Institute for Public Health and the Environment (RIVM), 3721 MA Bilthoven, The Netherlands; 3 Julius Center for Health Sciences and Primary Care, University Medical Center Utrecht, Utrecht University, 3508 TC Utrecht, The Netherlands

**Keywords:** COVID-19, BNT162b2, antibody

## Abstract

Vaccine-induced protection against severe COVID-19, hospitalization, and death is of the utmost importance, especially in the elderly. However, limited data are available on humoral immune responses following COVID-19 vaccination in the general population across a broad age range. We performed an integrated analysis of the effect of age, sex, and prior SARS-CoV-2 infection on Spike S1-specific (S1) IgG concentrations up to three months post-BNT162b2 (Pfizer/BioNTech; Comirnaty) vaccination. In total, 1735 persons, eligible for COVID-19 vaccination through the national program, were recruited from the general population (12 to 92 years old). Sixty percent were female, and the median vaccination interval was 35 days (interquartile range, IQR: 35–35). All participants had seroconverted to S1 one month after two vaccine doses. S1 IgG was higher in participants with a history of SARS-CoV-2 infection (median: 4535 BAU/mL, IQR: 2341–7205) compared to infection-naive persons (1842 BAU/mL, 1019–3116), *p* < 0.001. In infection-naive persons, linear mixed effects regression showed a strong negative association between age and S1 IgG (*p* < 0.001) across the entire age range. Females had higher S1 IgG than males (*p* < 0.001). In persons with an infection history, age nor sex was associated with S1 IgG concentrations. The lower magnitude of S1 antibodies in older persons following COVID-19 vaccination will affect long-term protection.

## 1. Introduction

Understanding and monitoring immune responses following COVID-19 vaccination is essential to protect the population against severe COVID-19. The mRNA BNT162b2 vaccine (Pfizer/BioNTech; Comirnaty) was the first COVID-19 vaccine to be approved by the FDA and EMA. As of July 2022, it was deployed in 164 countries around the world [1]. Generally, the vaccination strategy for BNT162b2 consists of a primary series of two vaccinations 3–6 weeks apart. Early studies of BNT162b2 demonstrated high vaccine efficacy against COVID-19 infection, after completing the full vaccination schedule [2]. This was subsequently confirmed by real-world data, when considering protection against severe COVID-19 [3,4].

The elderly population is at increased risk for severe COVID-19, hospitalization, and death. Age-related changes of the immune system, referred to as immunosenescence, contribute to an increased susceptibility to infectious diseases and reduced efficacy of vaccination in elderly persons [5,6]. Other research groups have shown impaired immune responses following COVID-19 vaccination, when comparing elderly nursing home residents to younger healthcare workers [7,8]. Aside from a difference in age range, nursing home residents and healthcare workers differ considerably from the general population. This is particularly true regarding the health status of nursing home residents and in terms of exposure to SARS-CoV-2 for healthcare workers. Further data concerning elderly people outside of nursing home settings are lacking [9], and data on the peak antibody concentrations post-vaccination in the general population as a whole remains limited [10,11]. Immunity within the general population as a whole tempers virus transmission, which further lowers the overall disease burden. Therefore, this information is essential for ongoing COVID-19 vaccination strategies, to be able to better protect vulnerable groups, like the community-dwelling elderly, against severe disease.

In the Netherlands, COVID-19 vaccinations were offered from early 2021 onwards. Initially, priority was given to frontline healthcare workers and individuals at high risk for severe COVID-19 due to long-term health conditions. For those at a high risk for severe COVID-19, national guidelines indicated the use of mRNA-1273 (Moderna; Spikevax) as the primary vaccination series. High risk groups were considered to be persons with class III obesity (BMI ≥ 40), Down syndrome, neurological conditions compromising breathing, or severe immunocompromising diseases such as haematological malignities, severe kidney failure and/or dialysis, history of organ, stem cell or bone marrow transplant, or severe primary immunodeficiencies. Subsequently, the general population was invited, based on descending age, to be vaccinated. Currently, COVID-19 vaccination is free of charge for everyone 5 years or older. The most commonly used vaccine for all ages is BNT162b2, except for those aged 60–64. Following national policies at the time, this group mostly received ChAdOx1 nCoV-19 (Jenner-Oxford; Vaxzevria, previously AstraZeneca) as their primary vaccination series.

In this article, we discuss COVID-19 serological findings across 1735 persons in the Dutch general population with an age range of 12 to 92 years following a primary series of BNT162b2. We determined the effect of age and sex on antibody acquisition one month following one and two doses of BNT162b2 and disaggregated the results by SARS-CoV-2 infection history. Subsequently, the decay in antibody concentrations from one to three months following two doses of BNT162b2 vaccination were analysed for persons ages 50 and over.

## 2. Materials and Methods

Data from two observational, longitudinal COVID-19 vaccination cohort studies were combined. Both studies had the same study design, with one focusing on adolescents and adults (12 to 60 years old) and the other on the ageing population (50+ years old). The primary end point for both studies was 28 days post-completion of a primary COVID-19 vaccination series. As such, participants were included in the study if they planned to receive a COVID-19 vaccination or had completed the primary vaccination series within the last 28 days. Data was collected between March and October 2021 (Table A1). For further details on study design, recruitment, and inclusion and exclusion criteria, see Appendix A.

Finger-prick blood samples and questionnaires were taken at four time points; prior to COVID-19 vaccination (Pre-vacc), 28 days after the first vaccination (Dose 1), 28 days after the second vaccination (Dose 2), and three months after the second vaccination (Month 3). Month 3 data was only available for the 50+ cohort. Questionnaires covered demographic factors (age, sex, date of sampling), COVID-19 vaccination information (number and dates of vaccination, vaccine brand), and SARS-CoV-2 testing information (if applicable; duration and type of COVID-19-related symptoms, if symptomatic; type and date of confirmatory testing by local health authorities, if symptomatic or asymptomatic). Finger-prick blood samples were self-collected in microtubes and returned by mail. Once received by the laboratory at the National Institute of Public Health and the Environment (RIVM), the Netherlands, serum was isolated from each sample by centrifuge and stored at −20 °C until sample processing.

Total immunoglobulin G (IgG) antibody concentrations to Spike S1 and Nucleoprotein were measured simultaneously using a bead-based assay, as previously described [12]. IgG concentrations were calibrated against the international standard for human anti-SARS-CoV-2 immunoglobulin (20/136 NIBSC standard) and expressed as binding antibody units per mL (BAU/mL) [13]. The threshold for seropositivity was set at 10.1 BAU/mL for Spike S1 [14] and 14.3 BAU/mL for Nucleoprotein [15]. Performance characteristics have been reported previously [15]: the specificity was 99% in pre-pandemic and 98% in post-pandemic negative controls for Spike S1 and 97% in post-pandemic negative controls for Nucleoprotein. The sensitivity for Spike S1 ranged from 90% for mild COVID-19 (i.e., symptomatic but not hospitalized) to 100% for patients hospitalized with COVID-19 between two weeks and two months after symptom onset. For Nucleoprotein, this was 85% and 100%, respectively.

All statistical analyses were performed in RStudio (R version 4.1.3, R Core Team, Vienna, Austria) [16]. Age in years at first vaccination was used. Persons who received one or two doses of BNT162b2 were selected. Persons who received BNT162b2 in a heterologous schedule were excluded from analysis. Samples were included if they were within −7 or +7 days from day 28 (Dose 1), −14 or +14 days from day 28 (Dose 2), and −14 or +14 days from three months (Month 3). Persons with serological evidence of a SARS-CoV-2 infection history (i.e., seropositive to Spike S1 at Pre-vacc) and/or a self-reported positive SARS-CoV-2 test performed by local health authorities prior to their first vaccination dose were analysed separately. SARS-CoV-2 confirmatory testing was available free of charge to anyone with COVID-19-related symptoms or those who were in close contact with someone who had tested positive according to national guidelines in the Netherlands. Persons who reported a positive SARS-CoV-2 test and/or were seropositive to Nucleoprotein after their first vaccination dose were excluded. Participants who were seronegative for Spike S1 but seropositive for Nucleoprotein at Pre-vacc were also excluded, as their infection status was considered inconclusive. Flowcharts of the number of participants and available samples are shown in Supplementary Figure S1.

The Mann–Whitney U test was used to compare antibody concentrations between infection-naive persons and those with a history of SARS-CoV-2 prior to vaccination. Not all participants in our study had measurements available at each time point. As such, for comparisons in paired samples (i.e., within each infection-history group but between time points) the Wilcoxon signed ranks test was performed on the subset of participants who had samples available at both time points, and the Mann–Whitney U test was performed on all available samples (assuming independence). If *p*-values differed between the two tests, the more conservative *p*-value was presented in the main text and figures, whilst the results for the alternative test were presented in the figure legend.

A linear mixed effects regression model with a random intercept per participant was used to determine the effect of time point (categorical; as described under sample collection), age (continuous), and sex (binary) on log10-transformed S1 IgG concentrations using lme4 (version 1.1.28 [17]). A model was created with these explanatory variables as well as an interaction term between time point and age and between time point and sex. Separate models were built for infection-naive persons and those with a history of SARS-CoV-2 infection. The Pre-vacc time point was not included in the model for infection-naive participants, as we assumed no effect of age and sex on seronegative IgG S1 concentrations. Backward stepwise model selection was performed, and the model with the lowest Akaike Information Criterion (AIC) was selected using the step function within lmerTest. For the 50+ cohort, a model was also built to determine the effect of age and sex on antibody levels at Dose 2 and Month 3. Lastly, two models were built to assess the effect of antibody level on antibody production following first vaccination, and then to assess antibody loss following completion of the primary vaccination series using paired samples. The former model used data from infection-naive participants with measurements at both Dose 1 and Dose 2, and the latter model used data from infection-naive participants with measurements at both Dose 2 and Month 3 (50+ cohort only). In addition to age and sex, we explored the effect of antibody concentrations produced at a previous time point on the fold-change to the next time point; e.g., the effect of IgG S1 concentrations at Dose 1 on the fold-change of antibody concentrations between Dose 1 and Dose 2. Fold-change was calculated with IgG S1 concentrations on the linear scale. Linear regression was conducted using age, sex, and IgG concentration as explanatory variables, as well as the interactions between these variables. Final model selection was based on the AIC, as described above.

Ethical approval was obtained through the Medical Research Ethics Committee Utrecht for both the 50+ population cohort (NL74843.041.21, EudraCT: 2021-001976-40), and for the adolescent and adult cohort (12–60 years) (NL76440.041.21, EudraCT: 2021-001357-31). All participants provided written informed consent. In the case of participants between ages 12 and 16, both parents also provided written informed consent.

## 3. Results

A total of 1735 BNT162b2-vaccinated participants were included and 4925 measurements were available: 1377 at Pre-vacc, 1429 at Dose 1, 1425 at Dose 2, and 694 at Month 3 (50+ cohort only). The number of participants per age group was: 144 for the 12–19 age group, 222 for the 20–29 age group, 306 for the 30–39 age group, 177 for the 40–49 age group, 255 for the 50–59 age group, 294 for the 60–69 age group, 259 for the 70–79 age group, and 78 for the 80–93 year-old age group (Supplementary Table S1). Older persons were less likely to have a pre-vaccination measurement, as the study commenced after the start of the national vaccination campaign, and vaccines were rolled out per age group from old to young. Fourteen participants dropped out of the study after enrolment (adolescent and adult cohort: two following their Pre-vacc measurement and three following Dose 1; ageing population cohort: five following Dose 1 and four following Dose 2). Overall, more females than males were included (1036; 60% vs. 687; 40%). The median interval between the two vaccination doses was 35 days (interquartile range, IQR: 35–35) and did not differ between age groups (Supplementary Table S1). For the 20–59 year-old age group, approximately 20% had a SARS-CoV-2 infection prior to vaccination, followed by 12% for the 12–19 age group, 5% for the 60–79 age group, and 0% for the 80+ age group (Supplementary Table S1).

### 3.1. Higher Spike S1-Specific IgG in Persons with a History of SARS-CoV-2 Infection

**Figure 1:**
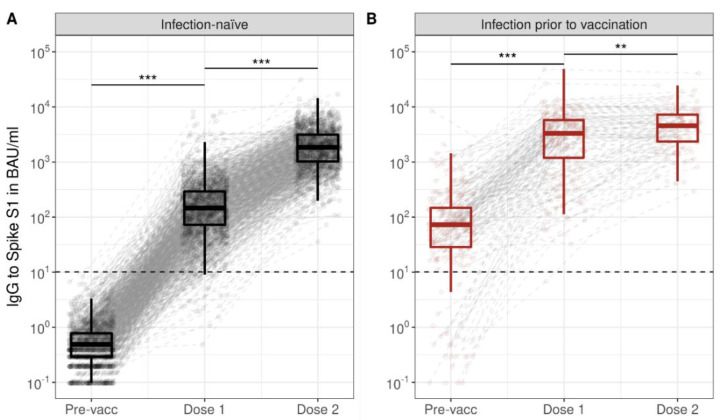
Spike S1-specific IgG kinetics following the primary series of BNT162b2 vaccination in infection-naive participants (**A**) and participants with a SARS-CoV-2 infection prior to vaccination (**B**). Boxplots show results for all participants at each time point, while dots and dashed grey lines show measurements and their trajectory between time points per participant. Results are shown for total of 1500 infection-naive participants and 235 participants with a SARS-CoV-2 infection history; measurements were not available at every time point for each participant. Spike S1 IgG concentrations were expressed in international binding antibody units (BAU) using the 20/136 NIBSC standard and were taken prior to vaccination (Pre-vacc), one month following the first (Dose 1), and one month following the second vaccination dose (Dose 2). The horizontal dashed line represents the threshold for seropositivity to Spike S1. Asterisks indicate *p*-values from Wilxocon Mann–Whitney U test (unpaired), when the Wilcoxon signed-rank test was performed in only paired samples all *p*-values were <0.001. IgG: immunoglobulin G; BAU: binding antibody units; ***: *p* < 0.001; **: *p* = 0.002.

At Dose 1, 97% (1204/1235) were seropositive among infection-naive persons, and 100% (193/194) among those who had an infection prior to vaccination. Seropositivity at Dose 1 decreased with age (Supplementary Table S2). Seropositivity was 100% at Dose 2, irrespective of age and prior infection status. For all ages, median S1 IgG in infection-naive increased from 0 BAU/mL Pre-vacc (IQR: 0–1), to 146 BAU/mL at Dose 1 (72–290; Pre-vacc vs. Dose 1: *p* < 0.001), and further to 1842 BAU/mL at Dose 2 (1019–3116; Dose 1 vs. Dose 2: *p* < 0.001), as shown in Figure 1. Persons who experienced a SARS-CoV-2 infection prior to vaccination increased from 73 BAU/mL Pre-vacc (29–147), to 3293 BAU/mL at Dose 1 (1191–5751; Pre-vacc vs. Dose 1: *p* < 0.001) and 4535 BAU/mL at Dose 2 (2341–7205; Dose 1 vs. Dose 2: *p* = 0.002); their S1 IgG was higher compared to infection-naive participants at each time point (*p* < 0.001).

### 3.2. Age Is a Strong Indicator of Spike S1-Specific IgG Acquisition following Vaccination

**Figure 2:**
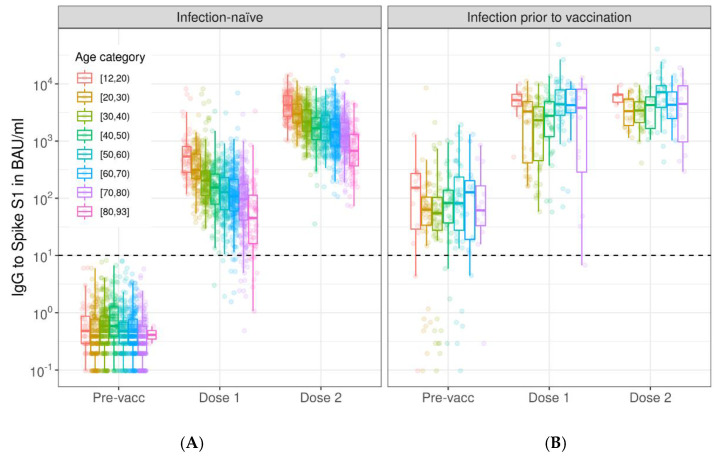
Spike S1-specific IgG kinetics by age category following the primary series of BNT162b2 vaccination in infection-naive participants (**A**) and participants with a SARS-CoV-2 infection prior to vaccination (**B**). Boxplots show results by age group at each time point, while dots show individual measurements. Results are shown for total of 1500 infection-naive participants and 235 participants with a SARS-CoV-2 infection history; measurements were not available at every time point for each participant (see Supplementary Table S2). Spike S1 IgG concentrations were expressed in international binding antibody units (BAU) using the 20/136 NIBSC standard and were taken prior to vaccination (Pre-vacc), one month following the first (Dose 1), and one month following the second vaccination dose (Dose 2). The horizontal dashed line represents the threshold for seropositivity to Spike S1. IgG: immunoglobulin G; BAU: binding antibody units.

**Figure 3:**
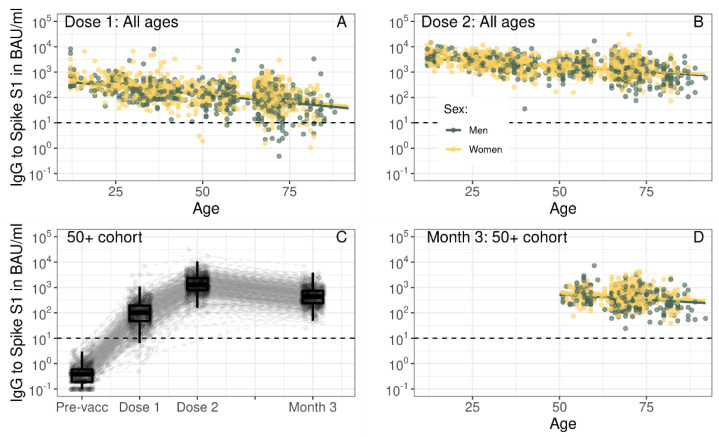
Spike S1-specific IgG by age in years per time point (**A**,**B**,**D**) and kinetics following the primary series of BNT162b2 vaccination in infection-naive participants up to three months following the second vaccination dose (**C**). In (**A**,**B**,**D**), fitted lines represent the linear association between IgG concentration and age from linear mixed effects regression results (Table 1), while dots represent individual measurements. Results are shown separately for males (green) and females (yellow). In (**C**), boxplots show results for all participants at each time point, while dots and dashed grey lines show measurements and their trajectory between time points per participant. In (**A**–**D**), Spike S1 IgG concentrations were expressed in international binding antibody units (BAU) using the 20/136 NIBSC standard and were taken prior to vaccination (Pre-vacc), one month following the first (Dose 1), one month following the second (Dose 2), or three months following the second vaccination dose (Month 3). In (**A**,**B**), results are shown for total of 1448 unique infection-naive participants across all ages with S1 IgG measurements available at Dose 1 and/or Dose 2, while in (**C**) results are shown for 749 unique infection-naive participants in the 50+ cohort and in (**D**) results are shown for 725 unique infection-naive participants in the 50+ cohort with measurements available at Dose 2 and/or Month 3. The horizontal dashed line represents the threshold for seropositivity to Spike S1. IgG: immunoglobulin G; BAU: binding antibody units.

BNT162b2-induced S1 IgG upon vaccination decreased in concentration with age, with the highest concentrations in the 12–19 year-old age group (Dose 1: median 526 BAU/mL, 282–770 and Dose 2: median 4198 BAU/mL, 2692–6131). We observed stepwise drops per age decade to a median concentration of 45 BAU/mL (16–113) for Dose 1 and a median concentration of 672 BAU/mL (366-1304) for Dose 2 in the 80–92 year-old age group (Figure 2, Supplementary Table S2). There was a strong negative association between age and IgG concentration at Dose 1 and Dose 2 (Table 1, Figure 3). The strength of the negative association between age and S1 IgG concentration decreased between Dose 1 and Dose 2 (coefficient of interaction term age*Dose 2: 0.004; *p* < 0.001), Table 1. Females had higher IgG S1 concentrations compared to males at Dose 1 (0.093; *p* < 0.001), but this difference was smaller at Dose 2 (coefficient of interaction term Female*Dose 2: −0.044; *p* = 0.048). Lower concentrations of S1 IgG at Dose 1 resulted in greater fold-change between the first and second vaccination doses (Supplementary Table S3). In persons with an infection history, age nor sex were associated with peak S1 IgG concentrations (Figure 2, Supplementary Figure S2, Supplementary Table S4).

### 3.3. Higher Peak Spike S1-Specific IgG Results in Greater Antibody Loss in Persons 50 Years and Over

For infection-naive persons in the 50+ cohort, median S1 IgG decreased from 1304 (772–2318) at Dose 2 to 440 (239–736) at Month 3, but all participants were still seropositive at Month 3 (Figure 3C, Supplementary Table S2). The strength of the negative association between age and S1 IgG was lower at Month 3 compared to Dose 2 (coefficient for interaction term age* Month 3: 0.004; *p* < 0.001, Figure 3D, Supplementary Table S5). The coefficient for higher S1 IgG in females compared to males remained the same between Dose 2 and Month 3 (0.102; *p* < 0.001). For antibody loss—i.e., the fold-change between one and three months following the second vaccination—higher concentrations of IgG S1 at Dose 2 resulted in a greater loss (Supplementary Table S6). For females, antibody loss was faster than for males at high concentrations of Dose 2 IgG S1 but slower at low concentrations of Dose 2 IgG S1. In persons with an infection history neither age nor sex were associated with S1 IgG concentrations at either post-vaccination time point (Supplementary Figure S2, Supplementary Table S5).

## 4. Discussion

This study shows high peak IgG responses to Spike S1 following two doses of BNT162b2 in infection-naive persons from the general population across a wide age range of 12 to 92 years. At the time of writing, this study is unique, as it included people of all ages from the general population who were sampled at fixed time points pre- and post-vaccination. This enabled direct comparison of S1 antibodies in adolescents, adults, and the elderly. The large numbers of participants in this study allowed the demonstration of a strong negative association between IgG S1 concentrations and age after the first dose of BNT162b2. This association was less pronounced but still present after the second dose.

Other studies have previously shown the inverse relationship between age and antibody concentrations following one dose of BNT162b2 in the infection-naive general population or healthcare workers [7,11]. However, observations of antibody concentrations following two doses are contradictory, with some studies reporting limited to no effect [7,9] and others reporting strong associations with age [10,18,19,20,21]. The pronounced age effect demonstrated in this study was still present one month after the second dose and, for the 50+ cohort, at three months following the second dose. A large study from the UK showed an age effect after one dose for both ChAdOx1 nCoV-19 (Oxford-AstraZeneca) and BNT162b2, but no effect of age after two doses when the vaccination interval was 8–12 weeks [10]. However, an age effect was present for BNT162b2, if the vaccination interval was three weeks. As the vaccination interval in our study was five weeks, these results combined indicate that receiving two vaccination doses and a wider vaccination interval are advantageous. Both act to nullify an age-dependent effect in peak humoral immune response in the case of a novel, primary vaccine like BNT162b2. The reported age effect in our study is in line with results from a recent pre-print, showing lower vaccine effectiveness against SARS-CoV-2 infection to the Delta and Omicron BA.1 variants with increasing age in the Netherlands [22]. Nevertheless, infection risk is determined by a complex interaction of infection pressure, behaviour, and the immune system’s response to invasion of the virus, which of course constitutes more than antibody concentrations alone.

Immunoageing in vaccine response is generally accepted; however, the decreasing trend in antibody responses from adolescent to older age is not largely reported, since COVID-19 vaccination introduced a new, primary vaccine for all ages. The higher IgG concentrations seen one month after the first dose in younger persons may be due not only to higher number of B cells but also to an overall superior B cell function, possibly involving a broader repertoire of naive B cells inversely related with age [23,24,25]. These B cells are more quickly activated in younger persons, upon exposure to the novel antigen introduced by the vaccine. Additionally, antigen uptake, processing, presentation, and signalling of innate cells, as well as recruiting T cell help, are more efficient in the younger versus the older age groups [26]. Together, this may contribute to higher antibody concentrations upon initial vaccination in younger persons. However, the increase in antibody concentrations induced by the second vaccine dose was greater for those with lower antibody levels after the first dose (seen in older persons). One explanation might be that high concentrations of circulating IgG—seen in younger persons—could partially blunt the response to the second vaccine dose. This would limit restimulation of antibody production following the second vaccine dose, particularly within the relatively short vaccination interval of five weeks in this study. Another explanation might be that peak antibody production was not yet achieved in older persons one month after the first vaccination dose. We also demonstrated that antibody loss between one and three months following the second dose was slower for those with lower peak antibody levels (seen in older persons) in the 50+ cohort. This could be explained by IgG half-life, which affects lower peak IgG concentrations in older persons less than higher peak IgG concentrations in younger persons [27], and by differences in underlying short-lived plasma cell responses and memory B cell formation across age ranges [23,24,25].

To date, most data on antibody responses following BNT162b2 vaccination in the elderly (80+) were derived from nursing home residents, the frailest group of older individuals [7,8]. The ageing population, however, shows a huge heterogeneity in health status, with early signs of ageing already occurring in the fifth decade of life (40–50 years) [28]. Diversity between individuals is mediated by a complex interplay of physiological and possibly pathological changes in metabolism, organ function, the innate and adaptive immune system, and differences in exposures to risk factors [26]. In nursing home residents ages 80 and over the seroconversion rate for Spike S1 following the primary series of BNT162b2 was 89% [8]. Comparatively our study showed 100% seropositivity in the elderly general population, outside of nursing home settings, up to three months following the second vaccine dose. Reassuringly, this implies that in a large part of the older general population immune response is better when compared to older persons in nursing homes. Similarly, Parry et al. also studied persons ages 80 and over in the general population and showed a seroconversion rate of 96% after two doses of BNT162b2 (*n* = 100) [9].

As previously described by others, we showed that females had slightly higher antibody concentrations than males following BNT162b2 vaccination [10,11,19]. This has been hypothesized to be due to sex steroid hormones or other genetic factors [5,6]. Moreover, in line with previous findings, we showed higher antibody concentrations in participants with a history of SARS-CoV-2 infection [10,11,29]. This finding has led to a national recommendation in The Netherlands of requiring a single dose to complete the primary vaccine series in persons with previous SARS-CoV-2 infection. We found that peak IgG S1 concentrations one month after the second dose were not affected by age or sex in persons with an infection history. However, in the 50+ cohort, at three months following the second dose, there was more variation in IgG concentrations as well as fewer observations available across a smaller age range. Still, the persistence of antibodies may differ with age and sex or for those receiving one or two doses following infection.

There were some limitations to our study. Firstly, it should be noted that persons 60–64 years old were underrepresented due to national guidelines to vaccinate with ChAdOx1 nCoV-19. Secondly, we did not have samples available from the adolescent and adult cohort (12–60 years old) at three months following the second vaccination dose. Additionally, we did not investigate the effect of comorbidities on BNT162b2-induced antibody concentrations in this study, as our aim was to determine the immune response in the general population as a whole. Other research groups have shown that seroconversion rates and antibody concentrations following mRNA vaccination are lower in patients with severe comorbidities when compared to healthy controls; these included patients with a history of solid organ transplantation [30], chronic kidney disease [31], and haematological malignancies [32]. Limited differences in antibody concentrations were seen in patients with lung disease, heart disease, autoimmune disease, diabetes, and hypertension when comparing BNT162b2-induced antibody concentrations to those in healthy controls [33]. However, antibody production was somewhat delayed for most of these patients. This was indicated by a larger difference in antibody concentrations between patient groups and healthy controls, after the first compared to the second vaccination. This delayed immune response has also been demonstrated in elderly nursing home populations, when compared to younger healthcare worker control groups [34]. Importantly, according to national guidelines in the Netherlands, groups at high risk for severe COVID-19 (i.e., those with severe comorbidities) were predominately vaccinated with mRNA-1273 (Moderna; Spikevax) during their primary series and were, therefore, unlikely to be included in our study population. Additionally, we studied circulating antibody concentrations, but, due to the large sample size, we have not been able to assess the neutralising capacity and avidity of these samples in vitro. A more in-depth focus on these antibody functionalities, in combination with cellular immune responses, will be further assessed longitudinally in subgroups of study participants in the future. Nevertheless, others have shown that high antibody concentrations are predictive of protection from COVID-19 infection and disease [10,35]. Lastly, we only investigated immune responses following BNT162b2 and not those following other COVID-19 vaccines. However, BNT162b2 is the most widely used vaccine for the primary series in the Netherlands, and the vaccine interval was consistent across all ages, which enabled direct comparison of vaccine responses across a broad age range.

## 5. Conclusions

We showed high peak IgG S1 concentrations post-BNT162b2 vaccination across all ages, including in older persons. Nevertheless, a strong negative association was demonstrated between these antibody concentrations and age. High concentrations of circulating antibodies are important for neutralizing SARS-CoV-2 infection, as recently exemplified by escape variants such as Omicron. Monitoring the persistence or decay of immune responses following vaccination is pivotal, specifically to evaluate antibody concentrations associated with immune protection in the elderly population. Such knowledge could support vaccination strategies to sustain optimal population immunity. Therefore, future work will focus on vaccine-induced SARS-CoV-2 humoral and cellular responses over time across age, infection history, number of received doses, and health status.

## Figures and Tables

**Table 1:** Linear mixed effects regression results for Spike S1 IgG concentrations up to one month following two doses of BNT162b2 for infection-naive persons. Model results for 1448 unique participants with S1 IgG measurements available at one month after the first (Dose 1) or second vaccination dose (Dose 2). Three persons reporting their sex as “other” were excluded from the model.

	Coefficient	95% CI	*p*-Value
Time point			
- Dose 1	Ref.		
- Dose 2	0.891	0.822, 0.960	<0.001
Age in years	−0.013	−0.014, −0.012	<0.001
Sex			
- Male	Ref.		
- Female	0.093	0.049, 0.136	<0.001
Age * Dose 2	0.004	0.003, 0.006	<0.001
Sex * Dose 2			
- Male * Dose 2	Ref.		
- Female * Dose 2	−0.044	−0.087, −0.000	0.049

* indicates an interaction term.

## Data Availability

The data presented in this study are available on request from the corresponding author. The data are not publicly available as the study is still ongoing.

## Supplementary Materials

The following supporting information can be downloaded at: https://www.mdpi.com/article/10.3390/vaccines10091480/s1. Figure S1: Flowchart of participants included in the study; Figure S2: Spike S1-specific IgG by age in years per time point in participants with a history of SARS-CoV-2 infection (A,B,D) and kinetics following the primary series of BNT162b2 vaccination up to three months following the second vaccination dose (C); Table S1: General characteristics of the study population one month following one and two doses of BNT162b2 by age category and SARS-CoV-2 infection status; Table S2: Spike S1 seropositivity and IgG concentrations one month following one and two doses of BNT162b2 by age category and SARS-CoV-2 infection status and at three months following the second dose for the 50+ cohort; Table S3: Linear regression results for fold-change in Spike S1 IgG concentrations at one month after the first and one month after the second dose in infection-naive participants across all ages; Table S4: Linear mixed effects regression results for Spike S1 IgG concentrations up to one month following two doses of BNT162b2 for participants with a SARS-CoV-2 infection history; Table S5: Linear mixed effects regression results for Spike S1 IgG concentrations one and three months following two doses of BNT162b2 by prior SARS-CoV-2 infection status in the 50+ cohort; Table S6: Linear regression results for fold-change in Spike S1 IgG concentrations at one month and three months following two doses of BNT162b2 in infection-naive participants from the 50+ cohort.

## Appendix A. Cohort Studies: Study Design, Recruitment, and Inclusion and Exclusion Criteria

Data from two COVID-19 vaccination cohort studies were combined. Details on the study designs and populations are described in detail below. Participants were included in the study if they planned to receive COVID-19 vaccination and were willing to participate. Those who had already received vaccination were also eligible if final recruitment occurred within the 28-day period following the second dose. Exclusion criteria are detailed below.

### Appendix A.1. Adolescent and Adult Cohort

Adolescents 12 to 17 years of age were invited to participate by simple random sampling from the National Population Register (Basis Registratie Personen; BRP). In total, 40,000 adolescents were invited, and the target was 150 participants. Adolescents were vaccinated with BNT162b2 (Pfizer/BioNTech; Comirnaty) according to Dutch national guidelines. Participants were excluded if they participated in a vaccination or medication clinical trial and/or were severely immunocompromised (Table A1).

Adults 18 to 60 years of age were invited to participate through multiple channels:

1. By simple random sampling from the BRP (*n* = 40,000);
2. Via participation in other studies by the Dutch National Institute of Public Health and the Environment (RIVM), so participants who consented to be contacted for additional research were invited;
3. By spontaneous applications from interested citizens.

The initial target was to include 150 participants per age group (18–29, 30–44, and 45–60) and vaccine type (BNT162b2 (Pfizer/BioNTech; Comirnaty), ChAdOx1 nCoV-19 (Jenner-Oxford; Vaxzevria, previously AstraZeneca), Janssen (Johnson & Johnson), and mRNA-1273 (Moderna; Spikevax)). However, BNT162b2 became the most widely used vaccine in the Netherlands, and, thus, no restrictions for enrolment were applied after inviting potential participants. Participants were excluded if they had participated in a vaccination or medication clinical trial and/or were severely immunocompromised (Table A1).

### Appendix A.2. 50+ Cohort

The Doetinchem Cohort Study (DCS) is an ongoing prospective study that began in 1987 in Doetinchem, in the eastern Netherlands [36,37]. The first study round in 1987 included a sex-stratified random sample of 20–59 years old inhabitants of the city of Doetinchem. Adults 50–90 years of age who had participated in round 6 (2013–2017) of DCS and consented to be contacted for additional research were invited to participate (*n* = 3147).

**Table A1:** Overview of study population, inclusion and exclusion criteria, and period of enrolment per cohort.

	Adolescent and Adult Cohort (18–60 Years)	50+ Cohort (50–92 Years)
Invited population	Random sample of national population register (*n* = 80,000)	Participated in round 6 of Doetinchem Cohort Study [36,37] (*n* = 3147)
	Participants in other studies by the Dutch National Institute of Public Health and the Environment (*n* = ~9500)	
	Spontaneous enrollment via website	
Inclusion criteria	Planning to receive/received COVID-19 vaccination *	Planning to receive/received COVID-19 vaccination *
	Able and willing to participate and sign IC	Able and willing to participate and sign IC
Exclusion criteria	Participation in a phase I/II/III preregistration vaccination trial or a phase I/II/III medicine (pre-registration) trial	None
	Belonging to a high-risk group for COVID-19 already studied in a risk group vaccination study that this study provides as a comparison for **	
	Any other immune deficiency through disease	
	Active or past immunosuppressive or immune modulating medication ***	
	Women who are pregnant or breastfeeding	
	Having (functional) asplenia	
	Receipt of blood products or immunoglobulin, within 3 months of study entry	
	Receipt of organ transplant not mentioned in a high-risk group list	
Period of enrolment	April to October 2021	March to August 2021

* Latest enrolment: within one month following second vaccination. ** Primary (inherited) immune deficiency, severely decreased kidney function (defined as chronic kidney disease stage 4 or 5; eGFR < 30), treatment by dialysis or recipient of a kidney transplant, pulmonary disease for which the patient will receive or has received a lung transplant, autoimmune disease (e.g., MS, rheumatoid arthritis, IBD, SLE, etc.), Down syndrome, (known) infection with Human Immunodeficiency Virus (HIV), cancer patients and patients with active cancer treatment (including hormone therapy), receipt of chemotherapy in the last 3 years and/or any history of cancer immune therapy, haematological patients, such as haematological malignancies (leukemia and lymphomas), myelodysplastic and -proliferative syndromes, hemoglobinopathies (sickle cell disease and thalassemia), or receipt of stem cell transplantation or cell therapy such as CAR T-cell therapy.*** For steroid treatment, the exclusion criteria were: receipt of any high-dose (≥20 mg of prednisone daily or equivalent) steroid treatment; daily corticosteroids (locally, incl. inhaled steroids, are acceptable) within 2 weeks of study entry; or repeated use of any high dose of corticosteroids (a dose of >30 mg of prednisone or equivalent per day for multiple days) in the recent past.
