# Supplementary material for: Reduced Antibody Acquisition with Increasing Age following Vaccination with BNT162b2: Results from Two Longitudinal Cohort Studies in The Netherlands
Source: Vaccines (Basel). 2022 Sep 6;10(9):1480. doi: 10.3390/vaccines10091480 (PMC9504637; doi:10.3390/vaccines10091480)
Supplement: Supplementary file 1 [file vaccines-10-01480-s001.zip › vaccines-1848395-supplementary.pdf]

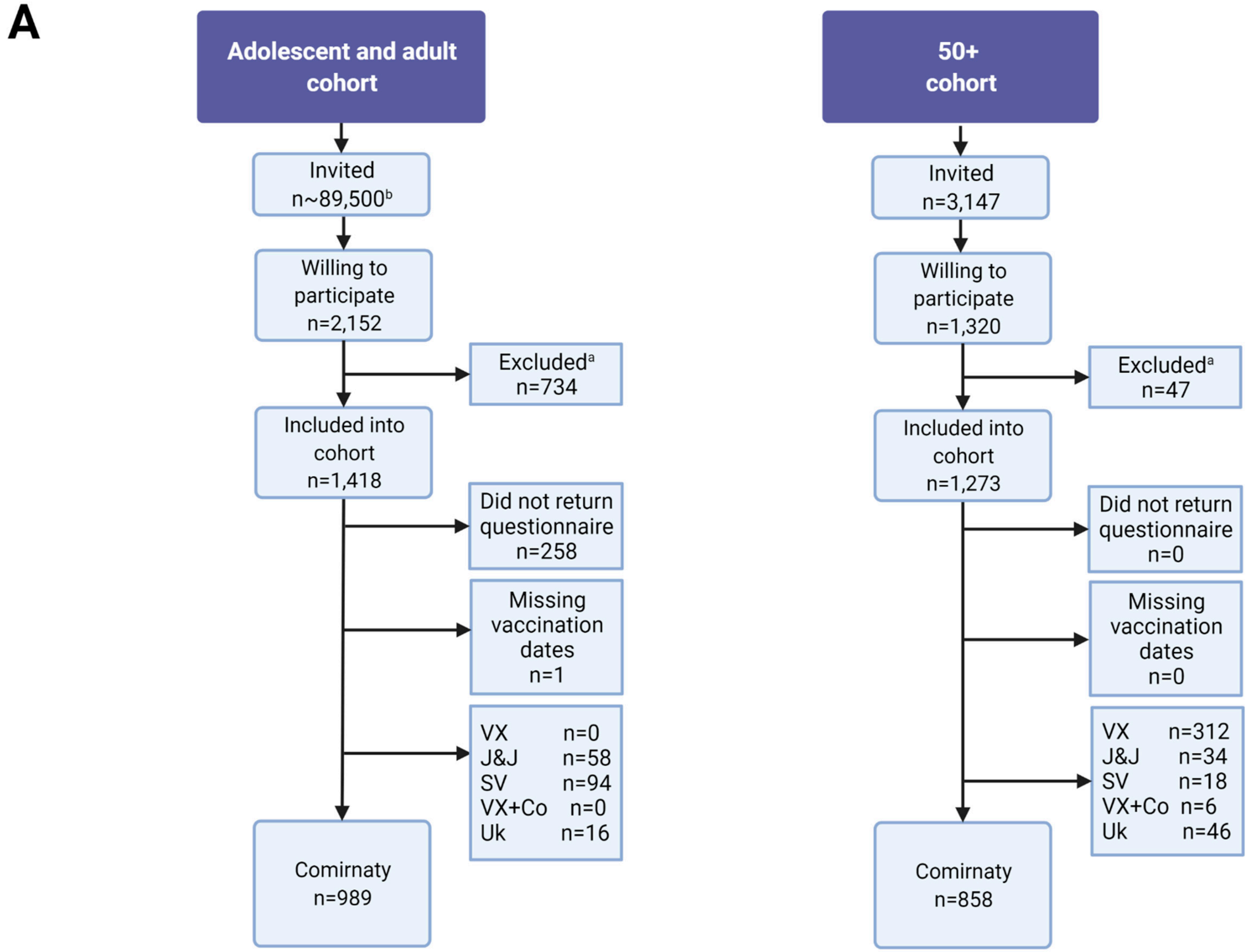
A
Adolescent and adult cohort
Invited n~89,500[b]
Willing to participate n=2,152
Excluded[a] n=734
Included into cohort n=1,418
Did not return questionnaire n=258
Missing vaccination dates n=1
VX n=0
J&J n=58
SV n=94
VX+Co n=0
Uk n=16
Comirnaty n=989
50+ cohort
Invited n=3,147
Willing to participate n=1,320
Excluded[a] n=47
Included into cohort n=1,273
Did not return questionnaire n=0
Missing vaccination dates n=0
VX n=312
J&J n=34
SV n=18
VX+Co n=6
Uk n=46
Comirnaty n=858

B

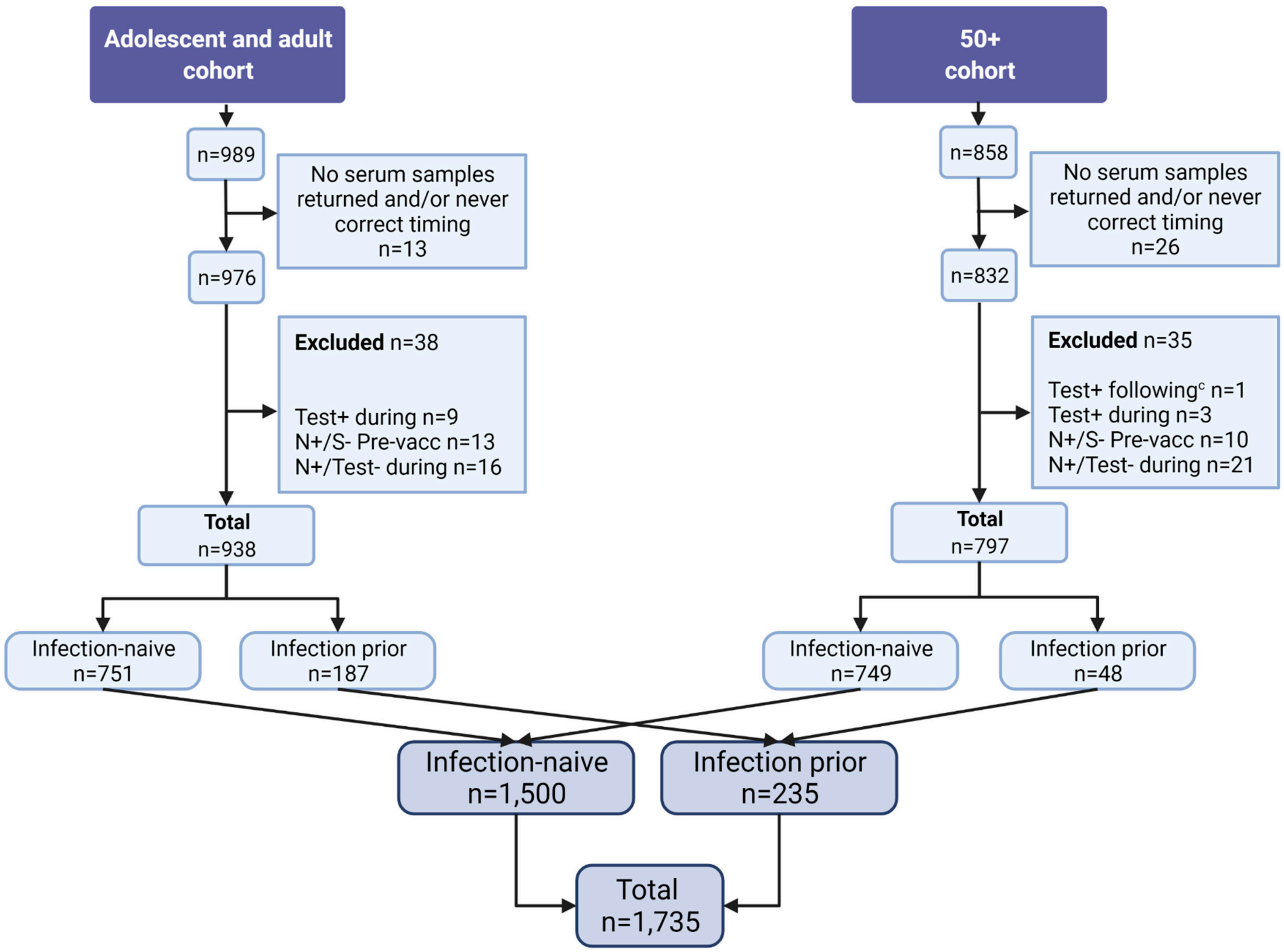

Adolescent and adult cohort
n=989
No serum samples returned and/or never correct timing n=13
n=976
Excluded n=38
Test+ during n=9
N+/S- Pre-vacc n=13
N+/Test- during n=16
Total n=938
Infection-naive n=751
Infection prior n=187
50+ cohort
n=858
No serum samples returned and/or never correct timing n=26
n=832
Excluded n=35
Test+ following[c] n=1
Test+ during n=3
N+/S- Pre-vacc n=10
N+/Test- during n=21
Total n=797
Infection-naive n=749
Infection prior n=48
Infection-naive n=1,500
Infection prior n=235
Total n=1,735

Supplementary Figure S1: Flowchart of participants included in the study. In (A) an overview is shown of the number of participants included into each cohort and who received Comirnaty vaccination. In (B) the number of participants without serum samples available and/or never returning a sample in the correct time window are shown as well as exclusions based on Nucleoprotein seroconversion or a positive SARS-CoV-2 test during the vaccination schedule, or those who were only seropositive to Nucleoprotein and not Spike S1 prior to vaccination as their infection-status was considered inconclusive. Participants were categorized into infection-naïve or those with a SARS-CoV-2 infection prior to vaccination (i.e., positive SARS-CoV-2 test and/or Spike S1 seropositive prior to vaccination). [a]Exclusion based on no further replies by participant after notification of willingness to participate, did not complete informed consent or – for the 12-17 and 18-60 cohorts only – based on exclusion criteria listed in Supplementary Table 1. [b]In addition to a total of 40,000 persons who were invited into the study by random sampling from the National Population Register (Basis Registratie Personen; BRP), approximately 9,500 participants who participated in prior studies by the Dutch National Institute of Public Health and the Environment (RIVM) and who consented to be contacted for additional research were invited as well as an unknown number of spontaneous enrollments from interested citizens. [c]For the 50+ cohort, participants with a positive SARS-CoV-2 test following vaccination were excluded as for this population measurements were analyzed up to three months following the second vaccination dose. VX: Vaxzevria; J&J: Janssen; SV: Spikevax; VX+Co: first dose Vaxzevria and second dose Comirnaty; Uk: unknown; Test+ during: positive SARS-CoV-2 test during primary vaccination schedule (antigen or PCR performed by local health authorities); N+/S-: seropositive to Nucleoprotein but seronegative to Spike S1 prior to vaccination; N+/Test-: seropositive to Nucleoprotein but no positive SARS-CoV-2 test reported during vaccination schedule; Test+ following: positive SARS-CoV-2 test following primary vaccination schedule (antigen or PCR performed by local health authorities).

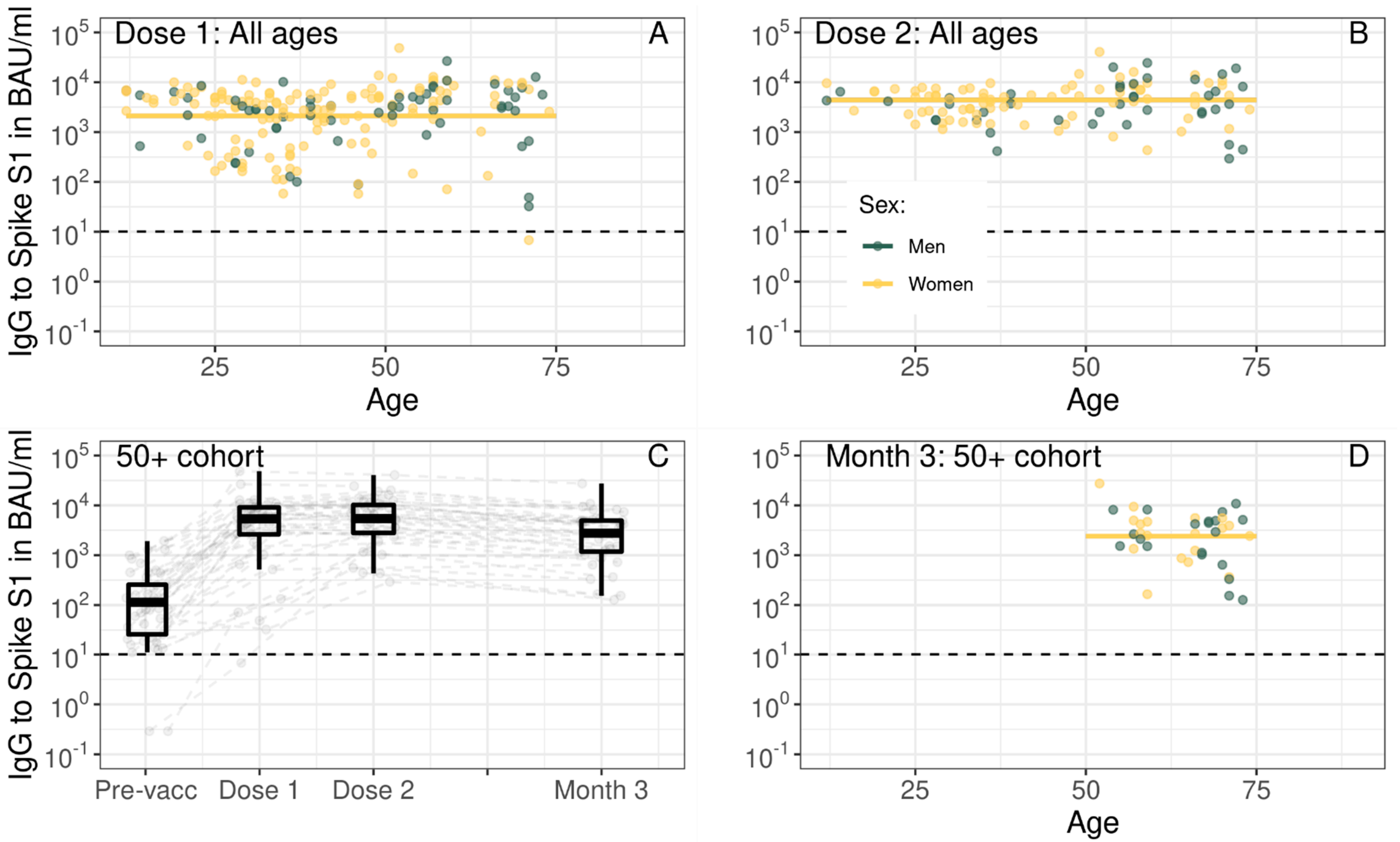

Supplementary Figure S2: Spike S1-specific IgG by age in years per timepoint in participants with a history of SARS-CoV-2 infection (A, B, D) and kinetics following the primary series of BNT162b2 vaccination up to three months following the second vaccination dose (C). In (A, B, D) fitted lines represent the linear association between IgG concentration and age from linear mixed effects regression results (see Supplementary Table 3 and Supplementary Table 4), while dots represent individual measurements. Results are shown separately for men

(green) and women (yellow). In (C) boxplots show results for all participants at each timepoint, while dots and dashed grey lines show measurements and their trajectory between timepoints per participant. In (A-D) IgG concentration measurements were expressed in international binding antibody units (BAU) using the 20/136 NIBSC standard and were taken prior to vaccination (Pre-vacc), one month following the first (Dose 1), one month following the second (Dose 2) or three months following the second vaccination dose (Month 3). In (A-B) results are shown for a total of 204 unique participants across all ages with S1 IgG measurements available at Dose 1 and/or Dose 2, while in (C) results are shown for 48 unique participants in the 50+ cohort and in (D) results are shown for 46 unique participants in the 50+ cohort with measurements available at Dose 2 and/or Month 3. The horizontal dashed line represents the threshold for seropositivity to Spike S1. The horizontal dashed line represents the threshold for seropositivity to Spike S1. IgG: immunoglobulin G; BAU: binding antibody units.

Supplementary Table S1: General characteristics of the study population one month following one and two doses of BNT162b2 by age category and SARS-CoV-2 infection status.

	12-19	20-29	30-39	40-49	50-59	60-69	70-79	80-93	All ages
N	144	222	306	177	255	294	259	78	1,735
**Infection-naïve**									
**N, %**	127, 88%	174, 78%	249, 81%	140, 79%	207, 81%	280, 95%	245, 95%	78, 100%	1,500, 86%
**Sex*, %**									
- Male	47, 37%	55, 32%	90, 36%	42, 30%	92, 44%	120, 43%	119, 49%	48, 62%	615, 41%
- Female	78, 61%	118, 68%	158, 64%	97, 69%	115, 56%	160, 57%	126, 51%	30, 38%	877, 59%
- Other	2, 2%	1, 1%	1, 0%	1, 1%	0, 0%	0, 0%	0, 0%	0, 0%	5, 0%
**Vaccination interval in days**									
- N with two doses	126, 99%	173, 99%	248, 100%	139, 99%	207, 100%	280, 100%	245, 100%	78, 100%	1,496, 100%
- Median	35	35	35	35	35	35	35	35	35
- IQR	23 – 35	35 – 35	35 – 36	35 – 36	35 – 35	35 – 35	35 – 35	35 – 35	35 – 35
**History of SARS-CoV-2 infection**									
**N, %**	17, 12%	48, 22%	57, 19%	37, 21%	48, 19%	14, 5%	14, 5%	0, 0%	235, 14%
**Sex*, %**									
- Male	5, 29%	12, 25%	15, 26%	7, 19%	21, 44%	7, 50%	8, 57%	N/A	75, 32%
- Female	12, 71%	36, 75%	42, 74%	30, 81%	27, 56%	7, 50%	6, 43%	N/A	160, 68%
- Other	0, 0%	0, 0%	0, 0%	0, 0%	0, 0%	0, 0%	0, 0%	N/A	0, 0%
**Vaccination interval**									

- N with two doses	9, 53%	30, 63%	37, 65%	18, 49%	39, 82%	13, 92%	14, 100%	N/A	160, 68%
- Median	28	35	35	35	35	35	35	N/A	35
- IQR	22 – 35	35 – 38	35 – 38	35 – 40	35 – 36	35 – 36	35 – 35	N/A	35 – 37

*In the adolescents and adult cohort study (ages 12 to 60), participants could indicate male, female or other. In the 50+ cohort, participants could only indicate male or female.

Supplementary Table S2: Spike S1 seropositivity and IgG concentrations one month following one and two doses of BNT162b2 by age category and SARS-CoV-2 infection status; and at three months following the second dose for the 50+ cohort.

	12-19	20-29	30-39	40-49	50-59	60-69	70-79	80-93	All ages
**Infection-naïve**									
**Seropositivity IgG to S1 (n/N)**									
- Dose 1	100% (56/56)	100% (148/148)	100% (207/207)	99% (124/125)	99% (176/178)	97% (238/245)	94% (206/219)	86% (49/57)	98% (1,204/1,235)
- Dose 2	100% (107/107)	100% (141/141)	100% (190/190)	100% (116/116)	100% (182/182)	100% (265/265)	100% (234/234)	100% (73/73)	100% (1,308/1,308)
- Month 3	N/A	N/A	N/A	N/A	100% (116/116)	100% (249/249)	100% (222/222)	100% (68/68)	100% (655/655)
**Median IgG to S1 (IQR)**									
- Dose 1	538 (284-805)	315 (221-594)	204 (112-301)	154 (79-265)	127 (63-231)	109 (60-215)	96 (42-176)	45 (16-113)	146 (72-290)
- Dose 2	4,218 (2,707-6,174)	2,972 (2,074-4,411)	2,177 (1,490-3,509)	1,673 (1,056-2,562)	1,765 (1,010-2,813)	1,435 (815-2,421)	1,238 (791-2,080)	672 (366-1,304)	1,842 (1,019-3,116)
- Month 3	N/A	N/A	N/A	N/A	486 (312-898)	456 (234-737)	446 (263-750)	210 (127-519)	440 (239-736)
**History of SARS-CoV-2 infection**									

**Seropositivity IgG to S1 (n/N)**									
- Pre-vacc	87% (13/15)	85% (41/48)	86% (49/57)	91% (32/35)	89% (41/46)	93% (13/14)	92% (11/12)	N/A	100% (200/227)
- Dose 1	100% (12/12)	100% (37/37)	100% (48/48)	100% (33/33)	100% (38/38)	100% (14/14)	92% (11/12)	N/A	100% (193/194)
- Dose 2	100% (6/6)	100% (18/18)	100% (28/28)	100% (12/12)	100% (29/29)	100% (12/12)	100% (12/12)	N/A	100% (117/117)
- Month 3	N/A	N/A	N/A	N/A	100% (15/15)	100% (12/12)	100% (12/12)	N/A	100% (39/39)
**Median IgG to S1 (IQR)**									
- Pre-vacc	153 (29-271)	63 (34-105)	55 (28-104)	83 (37-139)	82 (28-235)	128 (19-204)	62 (33-199)	N/A	73 (29-147)
- Dose 1	5,192 (4,085-6,561)	3,286 (416-4,884)	2,325 (457-3,941)	2,760 (1,190-4,862)	4,405 (2,814-7,810)	4,296 (3,112-8,090)	4,111 (401-8,085)	N/A	3,293 (1,191-5,751)
- Dose 2	6,446 (4,832-6,583)	3,404 (1,887-5,424)	3,425 (2,123-4,857)	4,336 (1,657-5,925)	7,119 (3,886-9,319)	4,375 (2,486-7,336)	4,531 (1,011-9,263)	N/A	4,535 (2,341-7,205)
- Month 3	N/A	N/A	N/A	N/A	2,774 (1,820-6,574)	2,827 (1,100-4,521)	2,967 (355-5,244)	N/A	2,774 (1,182-4,948)

Supplementary Table S3: Linear regression results for fold-change in Spike S1 IgG concentrations at one month after the first and one month after the second dose in infection-naïve participants across all ages. The dependent variable was the fold-change for antibody production which was calculated by dividing the IgG S1 concentration at Dose 2 by the IgG S1 concentration at Dose 1. Participants were only included if they had measurements available at both time points (n=1,089).

	**Coefficient**	**95% CI**	**p-value**
**IgG S1 concentration at Dose 1**	-43.914	-60.551,-27.276	<0.001
**Age in years**	-0.479	-0.794,-0.164	0.003
**Sex**			
- Male	Ref.		
- Female	86.976	40.710,133.241	<0.001
**IgG S1 concentration at Dose 1*Sex**			
- IgG S1 concentration at Dose 2*Male	Ref.		
- IgG S1 concentration at Dose 2*Female	-36.866	-58.229,-15.503	0.001

Supplementary Table S4: Linear mixed effects regression results for Spike S1 IgG concentrations up to one month following two doses of BNT162b2 for participants with a SARS-CoV-2 infection history. Results are shown for total of 204 unique participants across all ages with S1 IgG measurements available at Dose 1 and/or Dose 2.

	**Coefficient**	**95% CI**	**p-value**
**Timepoint**			
- Pre-vacc	-1.614	-1.726,-1.501	<0.001
- Dose 1	Ref.		
- Dose 2	0.260	0.122,0.398	<0.001

Supplementary Table S5: Linear mixed effects regression results for Spike S1 IgG concentrations one and three months following two doses of BNT162b2 by prior SARS-CoV-2 infection status in the 50+ cohort. Results are shown for total of 725 unique infection-naïve and 46 unique participants with a SARS-CoV-2 infection history in the 50+ cohort with measurements available at Dose 2 and/or Month 3.

	**Infection-naïve**			**History of SARS-CoV-2 infection**		
	**Coefficient**	**95% CI**	**p-value**	**Coefficient**	**95% CI**	**p-value**
**Timepoint**						
- Dose 2	Ref.			Ref.		
- Month 3	-0.792	-0.950,-0.635	<0.001	-0.299	-0.350,-0.248	<0.001
**Age in years**	-0.012	-0.016,-0.009	<0.001			
**Sex**						
- Male	Ref.					
- Female	0.102	0.053,0.151	<0.001			
**Age*Timepoint**						
- Age*Dose 2	Ref.					
- Age*Month 3	0.004	0.002,0.007	<0.001			

Supplementary Table S6: Linear regression results for fold-change in Spike S1 IgG concentrations at one month and three months following two doses of BNT162b2 in infection-naïve participants from the 50+ cohort. The dependent variable was the fold-change for antibody loss which was calculated by dividing the IgG S1 concentration at Month 3 by the IgG S1 concentration at Dose 2. Participants were only included if they had measurements available at both time points (n=633).

	**Coefficient**	**95% CI**	**p-value**
**IgG S1 concentration at Dose 1**	-0.099	-0.168,-0.031	0.004
**Age in years**	0.003	0.001,0.005	0.007
**Sex**			
- Male	Ref.		
- Female	0.322	0.041,0.603	0.025
**IgG S1 concentration at Dose 1*Sex**			
- IgG S1 concentration at Dose 2*Male	Ref.		
- IgG S1 concentration at Dose 2*Female	-0.091	-0.180,-0.001	0.048
